# Impact of Trivalent Arsenicals on Selenoprotein Synthesis

**DOI:** 10.1289/ehp.9440

**Published:** 2006-12-19

**Authors:** Denis Ganyc, Sarah Talbot, Fanta Konate, Sarah Jackson, Brian Schanen, William Cullen, William T. Self

**Affiliations:** 1 Department of Molecular Biology and Microbiology, Burnett College of Biomedical Science, University of Central Florida, Orlando, Florida, USA; 2 Department of Chemistry, University of British Columbia, Vancouver, British Columbia, Canada

**Keywords:** arsenite, dimethylarsinous acid, glutathione peroxidase, monomethylarsonous acid, selenite, thioredoxin reductase

## Abstract

**Background:**

Exposure to arsenic has been associated with development of skin, lung, bladder, liver, and kidney cancer. Recent evidence suggests that an increase in oxidative stress in cells treated with arsenicals represents the molecular mechanism behind arsenic-induced carcinogenesis. Selenium, in the form of selenocysteine, is necessary for the activity of several enzymes with a role in defense against reactive oxygen species. A mutual sparing effect between arsenic and selenium has been shown in animal studies when both metalloids are present in high concentrations.

**Objectives:**

To determine whether changes in selenoprotein synthesis may be an underlying mechanism behind arsenic-induced carcinogenesis, we analyzed the new synthesis of selenoproteins within cells after exposure to inorganic or methylated arsenicals using a human keratinocyte cell model.

**Results:**

Addition of arsenite to culture medium blocked new synthesis of selenoproteins when selenium was present in the form of selenite, and appeared to stimulate the use of serum-derived selenium. Monomethylarsonous acid (MMA^III^) treatment of cells, in contrast, did not block all new synthesis of selenoproteins but did result in an increase in cytosolic thioredoxin reductase (TrxR1) at both the mRNA and protein levels. MMA^III^ also reduced the new synthesis of cellular glutatione peroxidase (cGpx) and other smaller selenoproteins. Dimethylarsinous acid (DMA^III^) stimulated selenoprotein synthesis by an as yet unknown mechanism.

**Conclusions:**

These results suggest that arsenite and MMA^III^ are key metabolites that trigger higher levels of TrxR1, and both lead to a reduction in the expression of cGpx. Together these effects certainly could lead to carcinogenesis given the knowledge that many cancers have higher levels of TrxR, and reduced Gpx levels will reduce the cell’s ability to defend against reactive oxygen species. Based on these results, the impact of the trivalent arsenicals arsenite and MMA^III^ on selenoprotein synthesis may indeed represent a potential molecular mechanism for the higher rates of cancer observed in populations exposed to high levels of arsenic.

The metalloid selenium is required in the form of selenocysteine in several mammalian enzymes with roles in defense against reactive oxygen species (ROS). These include isoforms of thioredoxin reductase (TrxR), glutathione peroxidase (Gpx), and a selenium-dependent form of methionine sulfoxide reductase (SelR) (Arner and Holmgren 2000; Kryukov et al. 2002; Stadtman 2000; Tamura and Stadtman 1996). Selenocysteine is inserted via translation of a UGA codon, normally a stop codon, in a process that is well described in *Escherichia coli* and is currently being characterized in mammalian systems (Berry et al. 2001, 2002; de Jesus et al. 2006; Small-Howard et al. 2006). When selenium levels are reduced in the diet, selenoenzyme activities are similarly reduced (Hill et al. 1997; Lei et al. 1995). Selenium deficiency has been shown to result in dramatic increases in ROS with concomitant cell death (Saito et al. 2003). This cell death can be prevented by administration of vitamin E and other anti-oxidants, demonstrating that the primary need for selenium in a mammalian cell, at least in culture, is related to its role in enzymes involved in defense against ROS.

Thioredoxin reductase (TrxR), which catalyzes the NADPH-dependent reduction of oxidized thioredoxin, was first identified as a selenoprotein in 1996 by Tamura and Stadtman (Tamura and Stadtman 1996). Cellular glutathione peroxidase (cGpx), which catalyzes the glutathione-dependent reduction of hydroperoxides to their corresponding alcohol, was the first mammalian selenoprotein identified (Flohe et al. 1973). Several isoforms of Gpx with varying substrate specificity and expression patterns in different tissues have been identified (Brigelius-Flohe 1999). cGpx acts primarily on hydrogen peroxide, whereas phosphohydrolipid Gpx (PHGpx) has a broader specificity for cholesterol and phospholipid hydroperoxides. PHGpx also plays a critical role in reproduction and has been shown to regulate the activity of lipoxygenases (Straif et al. 2000; Ursini et al. 1999; Werz and Steinhilber 1996). As many as six isoforms of Gpx and four isoforms of TrxR have been uncovered in a recent *in silico* analysis of the completed human genome (Kryukov et al. 2003).

In stark contrast to selenium, exposure to the metalloid arsenic has been strongly linked to the development of cancer of the bladder, liver, lung, kidney, and skin, with skin as the primary target (Kitchin 2001). Exposure to elevated concentrations of arsenic is a public health crisis in areas of Bangladesh, Taiwan, and China. Although well established as a carcinogen, the mechanism of arsenic-induced carcinogenesis is not well defined at the molecular level. Many recent studies using cell culture model systems have demonstrated an increase in oxidative stress upon exposure to arsenicals (Kitchin and Ahmad 2003). Global gene expression analysis of primary keratinocytes treated with arsenite demonstrated a sustained upregulation of hemeoxygenase mRNA, a classic marker of oxidative stress (Rea et al. 2003). Arsenite was found to be more potent than the pentavalent arsenate in the induction of oxidative stress (Rea et al. 2003). The mRNA level of the selenoenzyme thioredoxin reductase was also increased up to 13-fold under these conditions; however, enzyme activities or protein levels of TrxR1 were not analyzed. Another study, also monitoring global gene expression in keratinocytes treated with arsenite, revealed increases in mRNA encoding thioredoxin and TrxR1 (Hamadeh et al. 2002). These studies assert that increased ROS generated upon long-term exposure to arsenic is the primary mechanism behind the increased rates of cancer in populations with elevated arsenic exposure. Although oxidative stress has been reported in several cases (Hamadeh et al. 2002; Kitchin and Ahmad 2003; Lee et al. 2004; Liao et al. 2004; Pi et al. 2003; Schwerdtle et al. 2003; Trouba et al. 2002), the mechanism for the increase in reactive oxygen species has yet to be elucidated.

Moxon first uncovered an interaction of arsenic and selenium in studies on the toxicity of selenium from seleniforous grains (Moxon 1938). Many animal studies have confirmed that a “mutual sparing effect” can be demonstrated when animals or cells in culture are treated with arsenic and selenium (Levander 1977; Styblo and Thomas 2001). A key paper published in 2000 (Gailer et al. 2000) identified selenobis(*S*-glutathionyl) arsinium ion as the major excretion product in rabbits administered arsenite and selenite. This likely uncovered the primary molecular mechanism behind the Moxon effect—which only relates to the co-administration of inorganic forms of arsenic and selenium. The direct analysis of the impact of arsenite and/or downstream methylated arsenic species on selenoprotein synthesis in either animal studies or tissue culture models has yet to be studied.

Although early toxicology studies ignored the potency of methylated forms of arsenic (Hirano et al. 2004; Kitchin and Ahmad 2003; Schwerdtle et al. 2003; Thomas et al. 2004), the available synthesis of trivalent forms of monomethylarsonous acid (MMA^III^) and dimethylarsinous acid (DMA^III^) now allows study of these compounds in cell culture model systems (Styblo et al. 2000). Indeed, these methylated forms have been shown to be more cytotoxic than inorganic arsenite and arsenate. The impact of these methylated arsenicals on the promotion of cancer is still poorly understood, and their potential interaction with selenium compounds has not been studied.

In this article we discuss the effects of inorganic and methylated trivalent arsenicals on selenoprotein synthesis using a keratinocyte cell model. This study was undertaken to determine the overall impact of arsenicals on selenoprotein synthesis.

## Materials and Methods

### Materials

Sodium selenite and sodium arsenite were purchased from Acros Organics (Geel, Belgium). MMA^III^ and DMA^III^ were obtained from W. Cullen, Department of Chemistry, University of British Columbia (Vancouver, Canada). ^75^Se radioisotope, in the form of selenite, was obtained from the University of Missouri Research Reactor (MURR, Columbia, MO). ^35^S-Methionine/cysteine labeling mix was from Amersham BioSciences (Piscataway, NJ).

### Cell culture

We cultured HaCat cells as a monolayer in Dulbecco’s modification of Eagle’s medium (DMEM) with l-glutamine, sodium pyruvate, and 4.5 g/L glucose supplemented with 10% fetal bovine serum (FBS), 100 μg/mL streptomycin, and 100 IU/mL penicillin (Mediatech, Herndon, VA). The cultures were incubated at 37°C with a humidified 5% carbon dioxide atmosphere to maintain proper pH. For cultivation in defined medium, cells were transitioned from DMEM to defined keratinocyte medium (DKM; Invitrogen, Carlsbad, CA) in 25% stepwise increments (e.g., 75% DMEM, 25% DKM first passage, 50% DMEM, 50% DKM second passage, and so forth). This conditioning allowed for optimal growth of the culture once growing in DKM without serum, as direct transition to DKM resulted in altered cell morphology and cell death.

We obtained the African green monkey kidney cell line, COS7, from the American Type Culture Collection (Manassas, VA). This cell line was used for reporter gene fusion assays, as the promoter for these constructs are driven by SV40 virus (Handy et al. 2005). COS7 were cultivated in DMEM supplemented with 10% FBS.

### Cell viability assay

To determine cytotoxicity of arsenicals, we cultured HaCat cells in 96-well dishes with approximately 2,500 cells per well. After one day of growth to allow for development of a healthy monolayer (70–80% confluent), arsenicals were added at varying concentrations and the cells were incubated for 24 hr. A tetrazolium dye, 3-[4,5-dimethylthiazol-2-yl]-2,5-diphenyl-tetrazolium bromide (MTT) was added (1.2 mM) and cells were subsequently incubated at 37°C with 5% CO_2_ in the dark for 4 hr. To solubilize the dye, we added 100 μL cell lysis solution (10% SDS, 5 mM HCl) to each well and incubated the plate overnight at 37°C. Absorbance of the soluble dye was determined at 570 nm in a Opsys MR microplate reader (Dynex Technologies, Chantilly, VA). We used a standard curve of known concentrations of cells (determined by direct cell counting using trypan blue in a haemocytometer) to validate the use of this assay as a representative measure of cell viability in this cell line.

### Radioisotope labeling of selenoproteins

The incorporation of selenium into seleno-proteins was monitored by labeling HaCat cells using ^75^Se in the form of selenite (University of Missouri). Unlabeled sodium selenite was added to a final concentration of 10 nM, and approximately 2 μCi of radioisotope was added to each culture (cultivated in 6-well cell culture dish). Under these growth conditions, only selenoproteins containing selenium in the form of selenocysteine are labeled. For analysis of general protein synthesis, we added 30 μCi of ^35^S in the form of methionine and cysteine (Redivue Pro-Mix ^35^S, Amersham BioSciences, San Francisco, CA) to HaCat cells in DMEM (low cysteine/methionine) with 10% FBS.

We harvested HaCat cells by treating the cells with 0.05% EDTA in Dulbecco’s phosphate-buffered saline (DPBS) without calcium/magnesium for 15 min. This EDTA solution was removed and replaced with trypsin–EDTA and further incubated at 37°C for 5 min. Cells released by treatment with trypsin were then isolated by centrifugation (500 × *g*), washed in PBS, and subsequently resuspended in 0.1 mL cell lysis buffer [50 mM tricine (pH 8.0), 0.1 mM benzamidine, 0.5 mM EDTA, and 1 mM DTT]. Cells were lysed by sonication for 5 sec using a Model 100 sonic dismembrator (Thermo Fisher Scientific, Pittsburgh, PA) at a power setting of 4 W. The resulting crude cell lysate was clarified by centrifugation at 13,000 × *g* for 5 min at 4°C. ^75^Se in cell extracts was detected using a Wallac Wizard Gamma Counter, Model 1470 (PerkinElmer, Wellesly, MA). ^35^S-labeled proteins in cell extracts were determined by liquid scintillation using a Packard TriCarb 2900TR counter (PerkinElmer). Protein concentration was determined by the method of Bradford using bovine serum albumin as a standard (Bradford 1976).

For radiolabeling of COS7, cells were seeded in 6-well tissue culture dishes in 0.5 mL DMEM with 10% FBS and cultivated for 24 hr at 37°C with 5% CO_2_. Cells were labeled with radioactive ^75^Se (2.5 μCi) and simultaneously treated with 0, 2, or 6 μM sodium arsenite. Once these compounds were added to the culture medium, cells were incubated for an additional 24 hr at 37°C with 5% CO_2_ before harvesting as described above, omitting the EDTA treatment prior to addition of trypsin.

### Immunoblot analysis of TrxR1 and cGpx

Polyclonal rabbit serum raised against the N-terminal peptide of human TrxR1 were a gift from T.C. Stadtman (National Heart, Lung, and Blood Institute/National Institutes of Health, Bethesda, MD). We obtained polyclonal sheep antibodies to cGpx from GeneTex, Inc. (San Antonio, TX). We treated HaCat cells with arsenite or MMA^III^ for 24 hr, then harvested the cells by treatment with trypsin as described above. Clarified cell extracts of HaCat cells (obtained after sonication as described above) were separated by SDS-PAGE (15%), subsequently transferred to polyvinylidene diflouride (PVDF) membrane, and blocked with Tris-buffered saline-Tween (0.2% Tween) containing 4% dry milk for 1 hr at 25°C. We incubated membranes with primary serum at 4°C for 16 hr at a dilution of 1:1000 in blocking buffer. After washing with TBS–Tween, we incubated the membrane with the appropriate secondary antibody conjugated with alkaline phosphatase at 25°C for 1 hr. The blot was developed using CDP-star chemiluminescent substrate (Applied Biosystems, Bedford, MA) and visualized on X-ray film.

### Real-time reverse transcriptase–polymerase chain reaction (RT-PCR) analysis of seleno-protein gene expression

We treated HaCat cells with arsenite or MMA^III^ for 24 hr, then harvested the cells by treating with trypsin as described above. Cells were subsequently washed with diethylpyrocarbonate (DEPC)-treated PBS. Total RNA was isolated using the ChargeSwitch Total RNA Cell kit (Invitrogen) and quantified by ultraviolet (UV)-visible spectrophotometry at 260 nm using an Agilent 8453 UV-Visible spectrophotometer (Agilent Technologies, Santa Clara, CA). Purified RNA (0.5 μg) was used as a template for the generation of cDNA using the iScript cDNA synthesis kit (Bio-Rad, Hercules, CA).

We performed real-time PCR amplification using the Bio-Rad i-Cycler (BioRad). Primer pairs used for analysis (forward, then reverse) were cGpx: 5′-GGGACTACACC CAGATGAA CG-3′ and 5′-CAAGGTG TTCCTCCCT CGTAG-3′; TrxR1: 5′-AGCTCAGT CCACCAATAGTGA-3′ and 5′-GGTATTT TTCCAGTCTTTTCAT-3′; β-actin: 5′-CATGTACGTTGCTATCCAGGC-3′ and 5′-CTCCTTAATGTCACGCA CGAT-3′. The level of transcripts for β-actin was used as an internal standard. BioRad iQ SYBR green supermix (Bio-Rad) was used for real-time PCR amplification with oligonucleotides at a concentration of 200 nM each. cDNA was diluted 1:100 before addition to the reaction mix. Reaction conditions (for β-actin and TrxR1 analysis) consisted of a single cycle at 95.0°C for 3 min; subsequent 40 cycles of 95.0°C for 10 sec, 55.0°C for 45 sec. For reactions using cGpx-specific primers the amplification was as follows: a single cycle at 94.0°C for 3 min; subsequent 40 cycles of 94.0°C for 30 sec, 55.0°C for 30 sec, and 70.0°C for 30 sec. Melt curve analysis was performed to confirm the presence of a single product. We calculated the efficiency of amplification for each target gene using a 10-fold dilution series of control cDNA. Relative expression of mRNA levels for TrxR1 and cGpx was calculated according to the Pfaffl method (Pfaffl 2001).

### Reporter gene fusion assays

A reporter gene fusion (UGA hSECIS S) previously described (Handy et al. 2005) was generously provided by D. Handy (Boston University School of Medicine, Boston, MA). COS7 cells were seeded at 80,000 cells per well in 24-well tissue culture plates in 1 mL DMEM with 10% FBS. After 24-hr incubation, cells were transfected with 250 ng luciferase (hSECIS) plasmid and 10 ng control Renilla plasmid (pRL-CMV; Promega, Madison, WI). Plasmids were pretreated with Plus reagent (Invitrogen). Lipofectamine 2000 was used for transfection of DNA in 200 μL Opti-Pro medium (Invitrogen). Cells were incubated with liposomes for 3 hr. DMEM supplemented with either 0.1 or 10% FBS (800 μL) was added, and the cells were subsequently treated with sodium selenite (10 or 50 nM) or 4 μM sodium arsenite for 24 hr. Transfection media was then removed and cells were cultivated for an additional 24 hr in DMEM with 0.1 or 10% FBS. After incubation, media was removed and cells were lysed in detergent lysis buffer supplied by the Dual-Luciferase Reporter Assay System (Promega). Luminescence was quantified as a ratio of Firefly luciferase/Renilla luciferase activity in cell extracts using an LMax luminometer (Molecular Devices, Sunnyvale, CA).

## Results

### Cytotoxicity of arsenicals in HaCat cells

**Figure 1:**
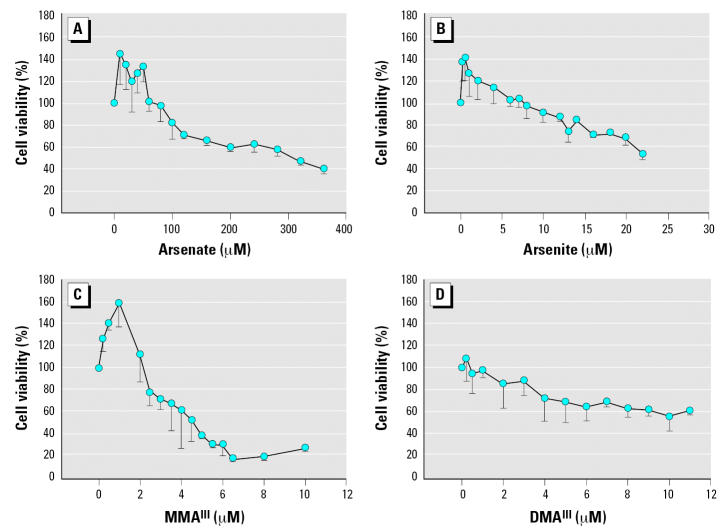
Cytotoxicity of trivalent arsenicals in HaCat cells cultured in DMEM containing 10% serum. HaCat cells were cultivated in the presence of arsenicals at concentrations indicated in each plot, noting that the scale is not identical. MTT dye reduction was assessed after incubating cells with arsenicals for 24 hr, and the absorbance obtained was compared with untreated cells to determine the relative percent cell viability. Data represent the mean of several experiments (minimum of three replicates) and the SD is plotted as error.

Before assessing the effects of arsenicals on selenium incorporation, we assessed the cytotoxicity of these compounds in HaCaT cultured in DMEM with serum (10%) using the MTT assay (Ohno and Abe 1991). These results are summarized in Figure 1. Pentavalent arsenicals were the least cytotoxic among the compounds tested. We observed a 50% reduction in cell viability when 375 μM arsenate was added to the culture medium. This is far from physiologically relevant concentrations of arsenicals, generally considered to be low micromolar or nanomolar levels when tested in cell culture systems to represent an environmental exposure (Hughes 2002). Similarly, cacodylic acid (dimethylarsinic acid - pentavalent) was not cytotoxic at levels < 400 μM (data not shown). It should be noted that lower concentrations (< 70 μM of arsenate) actually stimulated metabolism as determined by reduction of MTT dye. This was indeed the case for each arsenical tested, although this apparent proliferative effect was seen at far lower concentrations with other compounds (Figure 1).

In contrast to pentavalent compounds, trivalent arsenicals were far more cytotoxic. Arsenite reduced cell viability nearly 50% at 22 μM. MMA^III^, a trivalent methylated arsenical, was the most cytotoxic compound in this cell line with a 50% decrease in viability at 4 μM. DMA^III^ was less cytotoxic and resembled arsenite in the reduction of cell viability. These experiments were designed to determine the level of arsenical that does not significantly affect cell viability in 24 hr. These data guided our design of experiments to follow selenium incorporation using radiolabeled ^75^Se. The toxicity of these compounds has been reported previously, and our results are comparable to those seen in cell lines (primary keratinocytes) other than HaCat (Styblo et al. 2000).

### Selenium radioisotope studies to follow selenoenzyme synthesis in undefined media

To determine the impact trivalent arsenicals have on selenoprotein synthesis, we treated HaCat cells with several concentrations of each arsenical while introducing radiolabeled selenium (^75^Se) in the form of selenite to the culture medium. For this analysis, cells were treated with concentrations of arsenicals that did not significantly reduce cell viability (<50%) within 24 hr.

In control cells, the predominant selenoprotein bands corresponded with the expected molecular weight of TrxR1 (59 kDa) and cGpx, 22 kDa based on a molecular weight standard. This identification was confirmed with immunoblots (data not shown). Several potential isoforms of TrxR appear to be present, supported by *in silico* analysis that revealed several genes encoding TrxR-like enzymes (Kryukov et al. 2003). Other smaller selenoproteins are also present in HaCat and could be one of many small selenoproteins identified in the aforementioned genome analysis (Kryukov et al. 2003).

**Figure 2:**
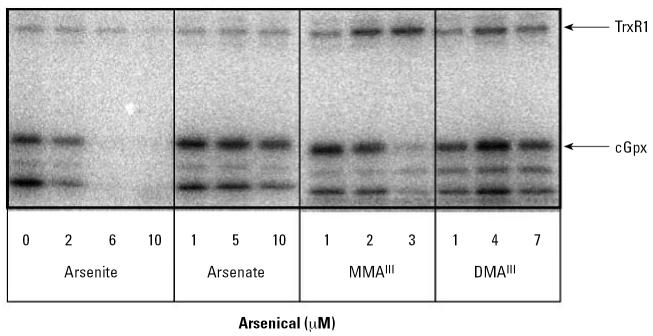
Effect of arsenicals on incorporation of selenium into selenoproteins in DMEM containing 10% serum. HaCat cells were cultivated in the presence of ^75^Se–selenite (10 nM) and treated with sodium arsenite, sodium arsenate, MMA^III^, or DMA^III^ at the concentrations indicated. Cells were harvested after 24-hr incubation with arsenicals and radioisotope selenium. Twenty-five micrograms of protein from crude cell extract were separated by 15% SDS-PAGE. Selenoproteins were identified by autoradiography using standard protein markers (molecular weight indicated at left of autoradiogram) based on Coommassie-stained gels before exposure.

When cells were treated with arsenite, we observed a clear concentration-dependent decrease in selenium incorporation into selenoproteins (Figure 2). The effect seems to be general inhibition as all the selenoproteins appear to decrease in parallel in the presence of increasing concentrations of arsenite. In contrast, cells treated with similar concentrations of arsenate (pentavalent) showed no significant changes. Treatment with similar concentrations of cacodylic acid also had no effect on selenoprotein synthesis (data not shown), and thus pentavalent compounds were not further tested.

In contrast to arsenite, treatment with low micromolar levels of MMA^III^ revealed a differential effect for incorporation of selenium. The level of radiolabeled TrxR1 increased, whereas selenium incorporation into cGpx and other small selenoproteins decreased. The differential effect of MMA^III^ on selenoprotein synthesis is certainly an intriguing result. Together these results show that MMA^III^ affected selenium metabolism in a fundamentally different manner than arsenite, demonstrating that each trivalent arsenical has a specific effect. Incubation of cells with DMA^III^ led to an increase in selenium incorporation into all selenoproteins at 4 μM, but this trend was reversed at higher concentrations (Figure 2). Similar experiments with cells harvested at later time points (e.g., 48 hr after addition of arsenite and radiolabeled selenium) exhibited similar results (data not shown), indicating that the keratinocytes cannot recover their ability to incorporate selenium into selenoproteins after brief adaptation to the arsenic exposure.

Arsenite does not inhibit general protein synthesis. To confirm that this inhibition was not simply due to a general inhibition of protein synthesis, we tested the incorporation of ^35^*S*-methionine/cysteine in the presence of varying concentrations of sodium arsenite. Although there was a slight increase in protein synthesis in the presence of low arsenite (200 nM), general protein synthesis was not significantly inhibited by arsenite (data not shown). It is possible that at low concentrations, arsenite (and other arsenicals) induces a proliferative effect on cells by increasing general protein synthesis. A recent study has also observed a similar proliferative effect in keratinocytes treated with these arsenicals (Mudipalli et al. 2005).

### Effect of arsenite is not cell-line specific

We examined the effect of treatment of other cell lines with arsenite to confirm that the inhibitory effect of arsenite was not isolated to one cell type. The well-studied epithelial cell line HeLa S3 and the mouse cell line NIH3T3 were chosen for these experiments. Addition of increasing concentrations of arsenite to either cell line in the presence of radiolabeled selenium also demonstrated a similar decrease in the incorporation of selenium into selenoproteins (data not shown). Thus, the apparent inhibition of selenium incorporation under these culture conditions appeared to be a general phenomenon for cells in culture. Because of the differential and pronounced effects of arsenite and MMA^III^ on selenoprotein synthesis, we further probed the effects of these compounds on expression of the genes encoding selenoenzymes in the HaCat model system.

### Treatment with arsenite or MMA^III^ increases mRNA levels encoding TrxR1

**Figure 3:**
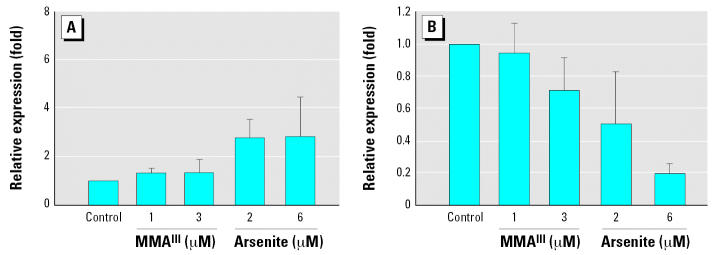
Treatment of HaCat with trivalent arsenicals results in increased expression of TrxR1 (*A*) and decreased expression of cGpx (*B*) when cells are treated in medium containing serum. Real-time RT-PCR analysis was used to determine the relative mRNA levels of transcripts encoding TrxR1 and cGpx. Fold induction is plotted versus control cultures (no arsenical addition). β-Actin levels were used as an internal control for quantitative RT-PCR analysis. See ”Materials and Methods” for details. Error bars represent mean ± SD for at least three independent RNA samples isolated from independent cultures.

Using real time RT-PCR we observed significantly higher levels of mRNA encoding TrxR1 upon treatment of cells with 2 or 6 μM arsenite (Figure 3A). In contrast, the level of cGpx mRNA was reduced by 80% compared with control cells when cultured with 6 μM arsenite. The reduction in cGpx mRNA was also concentration dependent (Figure 3B). MMA^III^ also slightly reduced the levels of mRNA encoding cGpx, and led to a similar increase in mRNA encoding TrxR1, yet in both cases the changes were not as dramatic. These results are in line with the selenium incorporation data from Figure 2 for MMA^III^ treatment—increases in TrxR1 levels and decreases in cGpx. However, for arsenite treatment, the results of real time RT-PCR analysis did not parallel the results shown in Figure 2. This suggests that arsenite treatment may somehow trigger the use of nonlabeled selenium present in the culture medium (serum-derived). Thus, we further assessed TrxR1 and cGpx protein levels to probe whether the apparent increase or decrease in mRNA encoding these enzymes also affected protein levels.

### Effect of trivalent arsenicals on TrxR1 and cGpx protein levels

**Figure 4:**
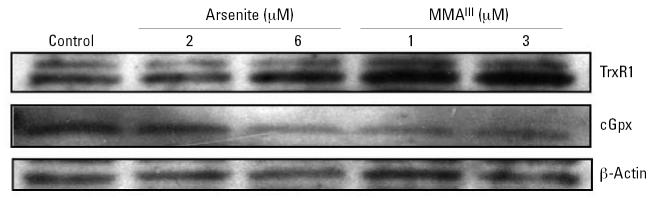
Measurement of TrxR1 and cGpx levels in cells treated with arsenite and MMA^III^. Immunoblot analysis of TrxR1, cGpx, and β-actin from cytosolic extracts of cells treated with arsenite and MMA^III^). Cytosolic proteins isolated from cells cultured in DMEM and 10% FBS and treated with arsenicals at the concentrations indicated were separated on a 4–20% SDS-PAGE gel, transferred to PVDF, and probed using polyclonal antibodies to TrxR1, cGpx, or β-actin. Immunoreactive bands were confirmed to correspond to radiolabeled proteins using authentic mouse liver TrxR1 and cGpx (data not shown).

Using antibodies specific for an N-terminal peptide of human TrxR1, we determined the amount of TrxR1 protein in cytosolic extracts after treatment of cells with MMA^III^ or arsenite for 24 hr. Figure 4 clearly shows that treatment with arsenite or MMA^III^ resulted in significant increases in TrxR1. Densitometry revealed a 50% increase in TrxR1 levels treated with 6 μM arsenite and a nearly 2-fold increase in TrxR1 levels in cells treated with either 1 or 3 μM MMA^III^. The amount of cGpx protein decreased under the same conditions (nearly 50% in arsenite-treated cells, based on densitometry), confirming that the impact of arsenicals on mRNA levels is correlated to the amount of these proteins produced. These results also show selenoproteins are still being produced in cells treated with arsenite or MMA^III^. Because the isotope labeling uses selenite, the uptake and metabolism of selenium sources other than selenite (serum-derived selenium sources) are apparently induced upon treatment with arsenite.

### Effects of trivalent arsenicals on seleno-protein synthesis in a serum-free defined culture medium

Uptake of either inorganic selenium or selenium derived for serum is poorly understood. Since selenoprotein P and a small molecule form of selenium, as yet unidentified, are present in FBS (Burk and Hill 2005), we determined the impact of arsenicals on selenoprotein synthesis in the absence of serum. We transitioned HaCat cells from DMEM with serum to a defined culture medium for keratinocytes (DKM, Invitrogen). This was carried out in a step-wise manner (see “Materials and Methods”). Once transitioned, cells grew at a slightly slower rate in DKM, but cell morphology was consistent with cells cultured with serum.

Before evaluating the impact of arsenicals on selenoprotein synthesis, the cytotoxicity of arsenicals was tested under these culture conditions. The cytotoxicity of arsenate and arsenite was similar to that seen in undefined media (data not shown), but MMA^III^ was slightly more cytotoxic. Even more toxic was DMA^III^, with a reduction of cell viability of 50% at approximately 7.5 μM. This was in contrast to very weak cytotoxicity of DMA^III^ in cells cultured with serum (Figure 1). This suggests that DMA^III^ may be interacting with components of serum, although this has yet to be tested directly.

**Figure 5:**
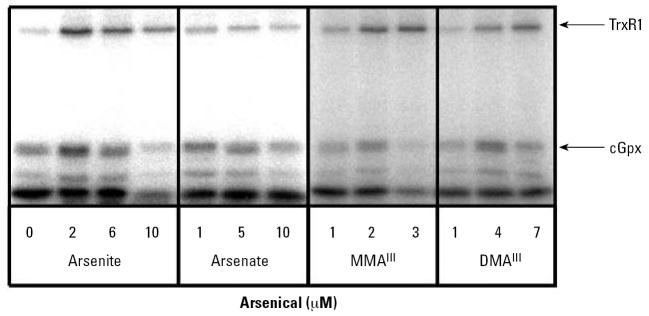
Impact of arsenicals on selenoprotein synthesis in HaCat cultured in defined keratinocyte medium (DKM). HaCat cells were cultivated in the presence of ^75^Se-selenite (10 nM) as in Figure 2 with the exception of being cultivated in DKM. Twenty-five micrograms of protein from crude cell extracts were separated by 15% SDS-PAGE.

Although the cytotoxicity was somewhat increased under these culture conditions, we analyzed selenoprotein synthesis by radioisotope labeling using the same conditions as in Figure 2 to enable a direct comparison. In contrast to the inhibition seen in Figure 2, arsenite actually stimulated the incorporation of radioisotope selenium into selenoproteins when arsenite was present at 2 and 6 μM in the culture medium. A higher concentration (10 μM) did result in an inhibitory effect, however, mimicking the inhibitory action of arsenite seen in Figure 2. Arsenate decreased slightly the level of radioisotope-labeled protein. The effects of both MMA^III^ and DMA^III^ on selenium incorporation were consistent with those seen in undefined medium (Figures 2 and 5). Clearly the greatest change in selenium labeling in DKM occurred upon treatment with arsenite.

### Treatment with arsenite or MMA^III^ in DKM results in induction of TrxR1 and decreases in cGpx expression

**Figure 6:**
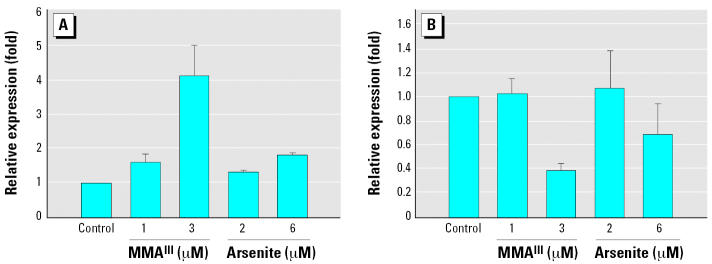
Treatment of HaCat with trivalent arsenicals results in increased expression of TrxR1 (*A*) and decreased levels of cGpx (*B*) mRNA when cultured in DKM. Real-time RT-PCR analysis was used to determine the relative mRNA levels of transcripts encoding TrxR1 and cGpx as in Figure 3. Error bars represent mean ± SD for at least three independent RNA samples isolated from independent cultures.

Again using real time RT-PCR, we analyzed the levels or mRNA encoding TrxR1 and cGpx in cells treated with MMA^III^ or arsenite but with cells cultured in DKM. As in undefined medium, the level of TrxR1 mRNA increased significantly, with an even more pronounced effect than that in cells cultured with serum. The increase in the level of mRNA encoding TrxR1 also appears to be concentration dependent with respect to the arsenical added to the culture medium (Figure 6A). Similar treatment with trivalent arsenicals also resulted in significant decreases in cGpx mRNA levels (Figure 6B). This indicates that the impact of trivalent arsenicals on mRNA levels encoding these two selenoenzymes is not affected by the presence or absence of serum.

**Figure 7:**
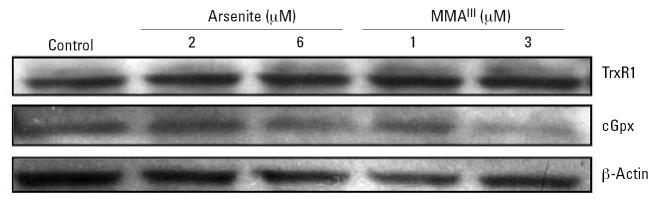
Arsenite and MMA^III^ inhibit new synthesis of TrxR1 and cGpx in defined medium (DKM). Immunoblot analysis of TrxR1, cGpx, and β-actin from cytosolic extracts of cells treated with arsenite and MMA^III^, as described in Figure 4.

Total selenoprotein synthesis is inhibited in cells treated with MMA^III^ or arsenite in DKM. Given that the levels of mRNA for TrxR1 are increased, we determined whether a significant increase in TrxR1 protein occurred using immunoblots. Indeed, Figure 7 clearly shows that the level of TrxR1 does not significantly decrease in cells treated with either arsenite or MMA^III^. However, cGpx levels do appear to be decreasing significantly (as much as 50% in cells treated with 3 μM MMA^III^ based on densitometry). This would imply that even in the presence of more abundant TrxR1 mRNA, the level of selenoprotein synthesis is stymied by these trivalent arsenicals in cells grown in the absence of serum. The decrease in cGpx is likely due to a more rapid rate of turnover relative to TrxR1, but this has yet to be shown. Nonetheless, these data strongly suggest that either MMA^III^ or arsenite can block the use of selenite for selenoprotein synthesis when cells are cultured in defined medium.

### Reporter gene fusion analysis of selenoprotein synthesis

**Figure 8:**
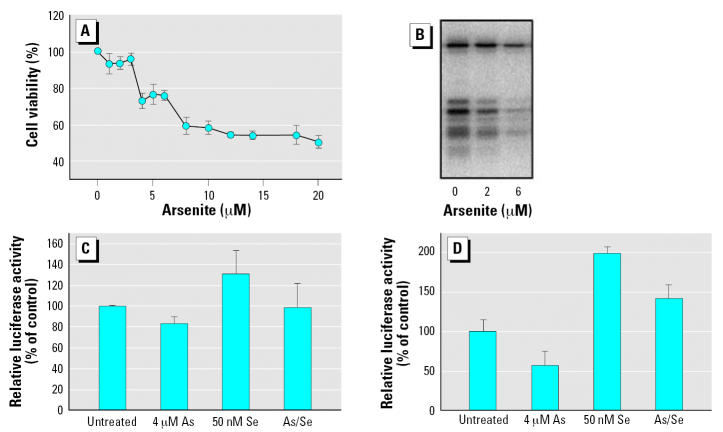
Arsenite reduces but does not eliminate readthrough of UGA codon in serum-containing medium. To analyze the cell’s ability to readthrough the UGA codon with the appropriate SECIS element, we used a gene reporter fusion described by Handy et al. (2005). (*A*) Cytotoxicity of arsenite in COS7 cells. MTT dye reduction was assessed after incubating cells with arsenicals for 24 hr, and the absorbance obtained was compared with untreated cells to determine the relative percent cell viability. Error bars represent mean ± SD for at least three independent cultures treated with arsenite. (*B*) Arsenite blocks incorporation of radiolabeled selenium in COS7 cells. Cells were cultivated in the presence of ^75^Se-selenite (10 nM) and treated with sodium arsenite at the concentrations indicated. Cells were harvested after 24-hr incubation, and 20 μg of protein from crude cell extract was separated by 12% SDS-PAGE. (*C*) Readthrough of UGA codon is resistant to arsenite. The gene reporter fusion denoted UGA SECIS/S (Handy et al. 2005) was transfected (250 ng) into COS7 cells cultured in DMEM with 10% serum. Transfection efficiency was determined by co-transfecting pRL-CMV encoding Renilla luciferase (Promega). Relative luciferase activity in control cells (no arsenicals) is taken as 100% readthrough. Selenium and/or arsenite was added to the culture medium before transfection at the concentrations indicated. (*D*) Readthrough of UGA codon determined in low-serum conditions (0.5% serum). Error bars represent mean ± SD for at least three independent transfected cultures.

Using a reporter gene fusion construct recently described by Handy (Handy et al. 2005), we assessed readthrough of the UGA codon [with appropriate selenocysteine insertion sequence (SECIS) element in the sense configuration] when COS7 cells were treated with arsenite. This analysis is limited to serum-containing media, as COS7 cells require serum for growth, but this would demonstrate the impact of arsenicals when serum selenium sources are provided. First, cytotoxicity of arsenite was assessed, and these results are shown in Figure 8A. Most notably, no stimulation of metabolic activity was observed in this cell line (African green monkey kidney cells). Low micromolar concentrations (1–3 μM) had little impact on cell viability, and a 50% reduction in cell viability was observed upon addition of 20 μM arsenite to the culture medium.

To confirm that the impact of arsenite would be similar to that seen in other cell types (HaCat, HeLa, NIH3T3), we treated COS7 with 2 or 6 μM arsenite in the presence of radioisotope selenium (Figure 8B). Arsenite again reduced incorporation of selenium into all selenoproteins in a concentration-dependent manner. Given these results, it was clear that arsenite is capable of blocking the use of selenite as the source of selenium in COS7 cells, even when present at concentrations that are not cytotoxic (as observed in HaCat and other cell lines).

The hSECIS/S (SECIS element in the sense orientation) reporter gene fusion was transfected into COS7 cells, and 3 hr after transfection (in serum-containing medium), arsenite was added at a concentration of 4 μM. Selenium was also added in the form of selenite to assess whether the readthrough was stimulated by addition of selenite, as previously reported (Handy et al. 2005). Addition of selenite alone increased relative luciferase activity, approximately 30% above that of control cells (Figure 8C). Treatment with arsenite alone reduced the readthrough of the UGA codon. Although this was statistically significant (*p* < 0.05), the level of luciferase activity decreased only 20%. When selenite and arsenite were both added, readthrough was inhibited about 20% by addition of 4 μM arsenite; however, this reduction was not statistically significant (Figure 8C). This was consistent with radioisotope labeling of keratinocytes (Figure 2A) when assessing incorporation of selenium from selenite. The basal level of UGA codon readthrough is likely due to selenium coming from sources in the serum, which is consistent with the data presented in Figures 2 and 5 for treatment with arsenite.

To test this possibility, COS7 cells were transfected in low serum conditions (0.1%) and treated with arsenite. Cells were conditioned for 24 hr in low-serum medium before transfection to eliminate residual serum-derived selenium in the cellular selenium pools. Again, arsenite reduced readthrough of the UGA codon, and this inhibition was more pronounced with nearly 50% reduction. Addition of selenite increased read-through, but arsenite continued to reduce selenium incorporation (Figure 8D). The more potent impact of arsenite in low serum conditions supports the hypothesis that arsenite blocks only selenium derived from selenite, and not that derived from serum.

**Figure 9:**
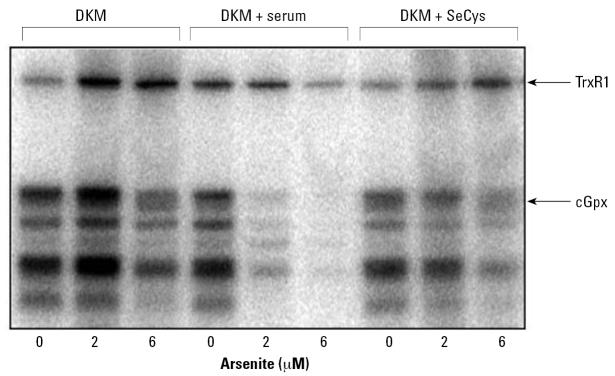
Arsenite treatment induces the use of selenium derived from serum but not from l-selenocysteine (SeCys). HaCat cells were cultivated in DKM as in Figure 5. Where indicated, FBS was added to 10%, or l-selenocystine was added to give 50 nM. Arsenite was added at the concentrations indicated below the autoradiogram, and cells were allowed to grow for 24 hr, as in the previous experiments shown in Figures 2 and 5.

Arsenite triggers the use of selenium derived from serum, but not l-selenocysteine. The cumulative results from radiolabeling, real-time RT-PCR, and reporter gene fusion (Figures 2, 5, 6, and 8) strongly suggest that treatment of cells with arsenite, when serum is present, stimulates the use of selenium from serum sources. Selenium is well established to be present in three forms in serum: selenoprotein-P, plasma Gpx, and a “small” molecule form of selenium that has yet to be characterized (Burk and Hill 2005; Schomburg et al. 2003). Because the transitioning of HaCat cells from serum-containing DMEM medium to DKM (with selenite as the only source of selenium) may have altered the cell’s response to arsenicals, we tested whether the addition of serum to DKM would recover the “inhibitory” effect of arsenite on selenium incorporation. When HaCat cells were cultured with 10% FBS in DKM, the response to arsenite mimics the results observed when cells were cultured with serum (DMEM; Figure 2). Although we could not test purified selenoprotein P, we separately assessed the impact of added l-selenocysteine to the culture medium in the presence and absence of arsenite. Surprisingly, the presence of arsenite in the culture medium still inhibited incorporation of selenium into all selenoproteins when “cold” l-selenocysteine was added instead of serum (Figure 9). Based on these results alone we cannot determine which component of serum might be used, but it is clear that exogenous addition of l-selenocysteine does not mimic the addition of serum-derived selenium.

## Discussion

There is a growing literature on the metabolic interactions of arsenic and selenium, yet none from this perspective. From the results presented in this report, we have obtained data that strongly suggest the use of serum-derived selenium is stimulated in cells in culture upon addition of arsenite to the culture medium. The uptake of selenium from serum, regardless of the source, is largely unknown. Thus, studies to determine the uptake of inorganic selenite versus selenium-derived from selenoprotein P may benefit from this analysis. Arsenite also caused an increase in mRNA levels for thioredoxin reductase, whether cultured in serum-containing media or defined media. This is likely due to activation of the antioxidant response element (ARE) in the TrxR1 promoter by Nrf2, as has been shown previously (Pi et al. 2003; Sakurai et al. 2005). It is clear from the immunoblot analysis that when serum is present, TrxR levels do increase, whereas cGpx does not. This is consistent with changes in the mRNA level. It cannot be ruled out, however, that the overall pool of selenium in culture is limiting, and competition between synthesis of higher levels of TrxR leads to a decrease in cGpx production.

Evidence presented in this study also demonstrates that methylated trivalent arsenicals have quite profound and different effects on selenoprotein synthesis compared with the arsenite. Treatment with MMA^III^strongly induced the synthesis of TrxR1, regardless of the source of selenium. This induction again may be due to activation by the Nrf2-dependent pathway, yet this is speculative, as no report in the literature has documented activation of the Nrf2 pathway using MMA^III^. MMA^III^ also did not block the use of selenium derived from selenite (assessed by radioisotope labeling), suggesting strongly that the addition of a single methyl group to trivalent arsenic alters the effect of arsenic on selenium metabolism. This is likely due to the inability of MMA^III^ to form a stable derivative with selenide, as has been shown previously for arsenite (Gailer et al. 2000, 2002b). It should be noted that both arsenite (including glutathiolated derivatives of arsenite) and MMA^III^ are potent inhibitors of enzymes containing vicinal dithiols (Delnomdedieu et al. 1993; Lin et al. 1999; Styblo and Thomas 1995). Clearly, the differential effects of these closely related arsenicals demonstrate that the observed changes in selenoprotein synthesis likely would not be attributed to binding to a vicinal dithiol.

Cancer cells have been shown to contain higher levels of TrxR, which is likely to support elevated needs for deoxyribonucleotide synthesis during proliferation (Kahlos et al. 2001; Soini et al. 2001). Indeed, several promising anticancer drugs have now been shown to target this enzyme with low side effects attributed to the differential expression of TrxR in tumors (Hashemy et al. 2006; Lu et al. 2006). Cells treated with MMA^III^ display a reduction in cGpx (at both mRNA and protein levels) as well as a strong induction of TrxR. If these changes are found to occur in other cell types and/or tissues, it likely would lead to increases in oxidative damage to protein, lipids and DNA while priming the cell for uncontrolled growth with higher TrxR activity. Thus, our results suggest that exposure of cells to MMA^III^ may be indeed a recipe for carcinogenesis. Two recent studies using bladder cell cultures also demonstrated oxidative stress upon treatment with MMA^III^ and arsenite, as well as transformation upon long-term exposure to MMA^III^ (Bredfeldt et al. 2006; Eblin et al. 2006). The impact that MMA^III^ has on other cell types, especially primary cells, with respect to selenoprotein synthesis will be the subject of future studies to determine if this effect is the key to this highly reactive compound’s carcinogenic potential.

DMA^III^ treatment resulted in increased incorporation of selenium into all selenoproteins yet had no effect on specific stimulation of TrxR1 and also did not block selenoprotein synthesis. It is not yet known why addition of a second methyl group would alter the effect on selenium metabolism, nor is it clear how administration of DMA^III^ resulted in higher levels of selenoprotein synthesis. DMA^III^ is also less toxic to cells in culture, likely because of the inability to target the vicinal dithiol of lipoic acid–containing enzymes of the Krebs cycle. It should be noted that a dimethyldiselenoarsinate anion (Gailer et al. 2002a) has been synthesized *in vitro*, but has yet to be identified in either animal or cell culture studies. Formation of this compound could possibly facilitate uptake of selenium, although this has yet to be tested.

In summary, the results of our study presented in this article demonstrate that trivalent arsenicals can significantly impact the metabolism of selenium and the expression and the synthesis of selenoproteins. Pentavalent arsenicals had negligible effects on selenoprotein synthesis. Identification of the molecular targets that each of these arsenicals acts upon within the cell to trigger these responses should add significantly to our understanding of the carcinogenic potential of arsenic.
